# tPA or not tPA? Lysis therapy in the setting of COVID‐19 and ECMO

**DOI:** 10.1002/jha2.430

**Published:** 2022-05-23

**Authors:** Anjelica Cromartie, Meghan Prin

**Affiliations:** ^1^ Department of GI Trauma, and Endocrine Surgery, University of Colorado Aurora Colorado USA; ^2^ Department of Anesthesiology University of Colorado Aurora Colorado USA

We read with interest the recent article entitled, “Using lysis therapy to treat five critically ill COVID‐19 patients who show echocardiographic criteria of right ventricular strain” by Mahdy et al., which described five patients who underwent lysis therapy for COVID‐19‐associated critical illness with respiratory failure and right heart strain. We find this topic especially relevant in the context of the global COVID‐19 pandemic, which has now claimed over 800,000 American lives and over 1.5 million worldwide. Critical illness secondary to COVID‐19 is associated with an inflammatory state and coagulopathy; the mechanisms underlying this state are still under investigation [1, 2, 3, 4]. There are multiple case reports describing adverse thrombotic events in this population [1, 2, 3, 4] and we are particularly interested in the authors’ report of Patient 5, who was receiving extracorporeal membranous oxygenation (ECMO) at the time of lysis therapy.

ECMO generally requires systemic anticoagulation due to a hypercoagulable state induced by contact of the patient's blood with the cannulas, but the choice of medication and the intensity of therapy vary in different settings and in patients with different medical conditions. The increased volume of distribution caused by the circuit also calls the standard dosing of alteplase into question. The balance between thrombotic events and bleeding complications is a delicate one without even accounting for the coagulopathy associated with COVID‐19.

Recently, we provided veno‐venous ECMO for a 47‐year‐old man with severe hypoxemia secondary to acute respiratory distress syndrome (ARDS) associated with COVID‐19 pneumonia. His hypoxemia was refractory to mechanical ventilation, neuromuscular blockade, and prone positioning. In addition, the patient had completed a course of dexamethasone and remdesivir as targeted therapy for COVID‐19. His medical history was significant for obesity, congenital solitary kidney, hypothyroidism, and hyperlipidemia. While on ECMO, he received therapeutic anticoagulation with unfractionated heparin.

On hospital day 17 (ICU day 13 and day 21 since symptom onset), our patient developed a massive pulmonary embolism with acute right ventricle (RV) failure (Figure 1). The patient's coagulation parameters on this day were as follows: PTT 102.3 s, INR 2, d‐dimer 85,940 FEU. He was too hemodynamically unstable to transfer for thrombectomy. After a multidisciplinary discussion, we gave intravenous alteplase (10 mg bolus over 1 min followed by 90 mg infusion over 2 h). Within minutes, he stabilized and there were no associated bleeding complications.

**FIGURE 1:**
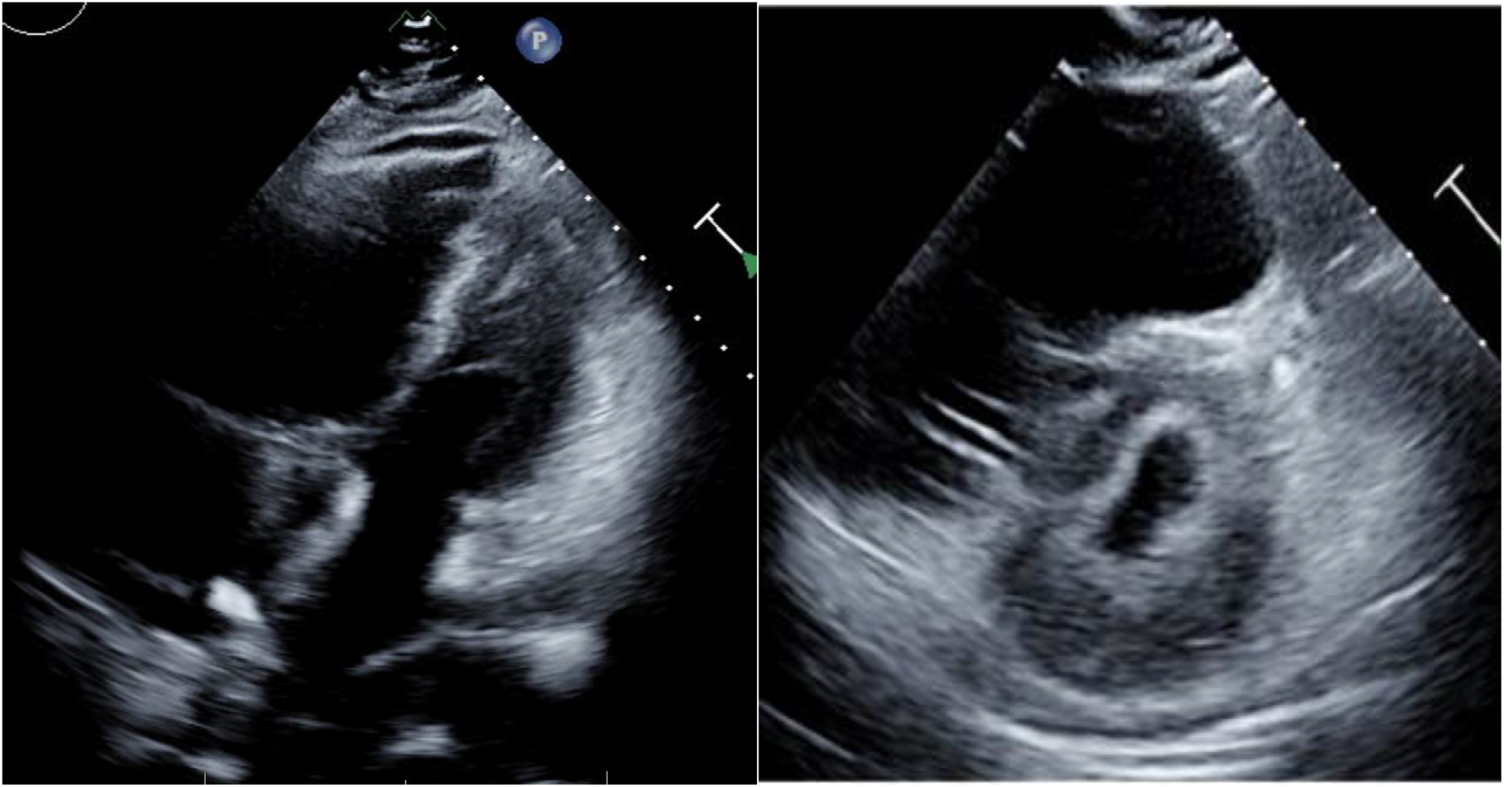
Four‐chamber apical and parasternal short views demonstrating severe dilation of right atrium and right ventricle (RV)

Anticoagulation decreases mortality in COVID‐19‐associated critical illness and is recommended while patients are on ECMO [2, 3, 4], but studies reporting thrombotic complications despite therapeutic heparin are common [5]. The ideal anticoagulation strategy for these patients is still unclear.

Prior to the pandemic, there were scant reports on the use of thrombolytics while on ECMO, leaving little guidance for clinicians at the bedside. We are curious if the authors of this case series are able to report more on Patient 5's coagulation studies, how they chose their alteplase dose, their consideration of bleeding risks, and if they have other recommendations for clinicians considering lytic therapy for patients with COVID‐19 disease requiring veno‐venous ECMO.

## CONFLICT OF INTEREST

The authors declare no conflict of interest.

## FUNDING INFORMATION

The authors received no specific funding for this work.
